# Doctors’ preferences in de-escalating DMARDs in rheumatoid arthritis: a discrete choice experiment

**DOI:** 10.1186/s13075-017-1287-z

**Published:** 2017-04-26

**Authors:** T. Martijn Kuijper, Riëtte Folmer, Elly A. Stolk, Johanna M. W. Hazes, Jolanda J. Luime

**Affiliations:** 1 Department of Rheumatology, Erasmus Medical Center, Room Na-609, PO Box 2040, 3000 CA Rotterdam, The Netherlands; 2 Institute for Medical Technology Assessment, Erasmus University, Rotterdam, The Netherlands

**Keywords:** Discrete choice experiment, Rheumatoid arthritis, Treatment de-escalation, Preferences

## Abstract

**Background:**

Current guidelines suggest reduction of DMARDs can be considered in RA patients in remission. Objectives were (1) to estimate the relative importance of patient characteristics rheumatologists consider in their decision to de-escalate (2) to assess whether heterogeneity exists among rheumatologists with respect to de-escalation and (3) to identify the preferred de-escalation strategy.

**Methods:**

A discrete choice experiment (DCE) was conducted. All rheumatologists and trainees in The Netherlands were invited to participate. A conditional logit model was estimated to assess overall preference for de-escalation and its determinants. Heterogeneity was estimated by latent class analysis.

**Results:**

The DCE questionnaire was completed by 156 doctors. This questionnaire was constructed using the results of semi-structured interviews with 12 rheumatologists that identified five patient characteristics relevant for de-escalation: number of swollen joints (SJC), presence of DAS remission/low disease activity (LDA), patient history, duration of remission/LDA and patient willingness to de-escalate DMARDs. Overall SJC and patient history were most important. Latent class analysis revealed five subgroups of doctors, showing differences regarding willingness to de-escalate and relative importance of patient characteristics. De-escalation of the TNF inhibitor rather than methotrexate first was the most preferred strategy.

**Conclusions:**

Rheumatologists are not uniform in their decision on whom to de-escalate. Differences emerged in which characteristics they traded off resulting in five subgroups: those that taper (1) always, (2) in absence of swollen joints, (3) in absence of swollen joints and presence of favorable patient history, (4) in DAS remission and favorable patient history, and (5) taking into account all factors.

**Electronic supplementary material:**

The online version of this article (doi:10.1186/s13075-017-1287-z) contains supplementary material, which is available to authorized users.

## Background

Treatment of rheumatoid arthritis (RA) has advanced greatly during the past decades. The introduction of combination therapy with disease-modifying anti-rheumatic drugs (DMARDs), the recognition of early, tightly controlled treatment, and the introduction of biologic agents have contributed to improved outcomes for patients suffering from RA [1]. With intensive use of (a combination of) DMARDs, a state of low disease activity (LDA) or remission can be achieved by many patients while preventing erosions and functional impairment [2, 3]. Although DMARD therapy is essential to obtain disease control, continuous use comes with several disadvantages. Apart from obvious drawbacks such as drug toxicity and side effects, medication use by itself may be perceived as burdensome and unhealthy by patients. Hence many patients view medication use as a necessary burden and wish to minimize its use. Also medication costs, especially for expensive biological treatments, are of increasing concern for governments. From this viewpoint tapering or discontinuation of DMARDs is preferable once disease control has been obtained.

Current guidelines suggest that reduction of biological (b)DMARDs can be considered, especially if this treatment is combined with a conventional synthetic (cs)DMARD, once sustained remission has been achieved and glucocorticoids have been tapered first [4, 5]. In addition, guidelines state that a cautious reduction of csDMARDs could be considered, as a shared decision between patient and physician, after glucocorticoids and bDMARDs have been successfully withdrawn [5]. Furthermore, a general recommendation is included that, apart from disease activity, other factors should be taken into account such as progression of structural damage, comorbidities, and safety issues [5].

Indeed, evidence from a range of clinical studies suggests that de-escalation of DMARDs is feasible in a large number of patients in LDA or disease remission [6–8]. However, to date there is no standardized way to determine the patient for whom de-escalation of DMARD therapy is appropriate [8]. Also, adherence of rheumatologists to the guidelines that are currently available for de-escalation may not be optimal [9]. Therefore large differences are expected to exist between rheumatologists with respect to whether, when, and in which patients they will de-escalate therapy. Obtaining insight in these differences may assist in future guideline development and guide further research into this topic. In the assessment whether a patient is a suitable candidate for treatment de-escalation, rheumatologists weigh several patient characteristics together at the same time. Hence a simple questionnaire in which the importance of patient characteristics is rated separately does not reflect a real-life decision. A discrete choice experiment (DCE) is a method that allows for the analysis of such complex decisions. It does so by presenting a series of “choice tasks” to the participant. Each choice task consists of two hypothetical patients with varying characteristics. The participant must then choose the patient with the most favorable combination of characteristics. By analyzing the choices participants made based on the characteristics, the relative importance of patient characteristics on the decision to de-escalate treatment can be assessed. A technique very similar to that of a discrete choice experiment was used in the process of the development of the American College of Rheumatology/European League Against Rheumatism (ACR/EULAR) 2010 criteria for RA [10].

Objectives of this study were (1) to estimate the relative importance of patient characteristics rheumatologists consider in their decision to de-escalate medication, (2) to quantify how these characteristics influence doctor’s preferences for de-escalating DMARDs, (3) to assess whether heterogeneity exists among rheumatologists with respect to their preference for de-escalation and the patient characteristics influencing this decision, and (4) to identify the most preferred de-escalation strategy.

## Methods

### DCE

In a DCE it is assumed that the analyzing process leading to a decision to medically intervene, such as de-escalation of medication, can be described by features relevant for making that decision (patient and disease characteristics) [11, 12]. In the case of a rheumatologist deciding whether to de-escalate DMARDs for a certain patient, these characteristics are likely disease related (e.g., presence of swollen joints indicating active disease). Each characteristic can then be further described by specific variants or levels (e.g., presence of no, one or two swollen joints) [11, 12]. Another assumption is that an individual’s preference for the intervention is determined by the levels of these characteristics [11, 12]. By offering a series of choices between two options with different characteristics (patients with different characteristics), the relative importance of the characteristics on the decision can be determined [11].

### Questionnaire design

To identify patient characteristics that determine the decision-making for DMARD de-escalation, 12 rheumatologists were randomly selected for interviews stratified by region and type of hospital (university or general). A semi-structured interview schedule (Additional file 1) was designed based on characteristics identified by pilot interviews of rheumatologists and literature [7]. During the semi-structured interviews, rheumatologists were questioned by telephone about their personal opinion and attitude with respect to DMARD de-escalation. The number of interviews was deemed sufficient as no new themes were mentioned during the final interviews, and therefore no other rheumatologists were approached. Based on the interviews, five patient characteristics with corresponding levels were developed (Table 1).

**Table 1:** Choice task example

	Patient A	Patient B
Duration of remission^1^	6 months	1 year
Patient preference for de-escalation at the start of the consult^2^	Patient is not willing to de-escalate	Patient is willing to de-escalate
Number of swollen joints^3^	1	2
DAS28^4^	≤3.2	<2.6
Medical history^5^	Difficult to accomplish remission	Easy to accomplish remission
	Non-erosive	Erosive

Participants were required to choose the patient they deemed most suitable for de-escalation or neither (opt-out). For each choice task patient characteristics were varied by assigning different levels. Possible levels are indicated in the subscript. All patients were assumed to use the combination of methotrexate 20–25 mg/week and a TNF blocker

*DAS* Disease Activity Score

^1^Levels were “6 months” and “12 months”

^2^Levels were “patient is not willing to de-escalate” and “patient is willing to de-escalate”

^3^Levels were “0”, “1” and “2”

^4^Levels were “≤3.2” and “<2.6”

^5^Levels were “difficult to accomplish remission, erosive”, “difficult to accomplish remission, non-erosive”, “easy to accomplish remission, erosive” and “easy to accomplish remission, non-erosive”

### Study design and questionnaire

A questionnaire containing 16 choice tasks, each consisting of two hypothetical patients, was deemed feasible to complete by rheumatologists attending the annual convention of the Dutch Society of Rheumatology (Nederlandse Vereniging voor Reumatologie, NVR). All hypothetical patients were assumed to use the combination of methotrexate 20–25 mg/week and a tumor necrosis factor (TNF) blocker, without additional glucocorticoids. In order to gain as much information as possible (enhancing precision) from a limited number of choice sets and sample size, computer software using experimental design theory (Ngene 1.1.2, 2014 ChoiceMetrics software, Sydney, NSW, Australia) was used to generate the most efficient sets of choices given the characteristics as defined previously (optimized for a main effect model with full dummy specification). To further optimize this process, priors were chosen reflecting the expected direction of characteristic levels on preference (Additional file 2), but not imposing any strong assumptions about the weight of each characteristic in the decision that might (dis)favor identification of some effects over others.

Choice sets consisted of two hypothetical patients and an opt-out option (Additional file 3). From each choice set, rheumatologists were asked to choose the patient they deemed most suitable to de-escalate DMARDs. The opt-out option was included in case respondents deemed neither patient suitable for de-escalation, resembling real-life decision-making. Of note, only after rheumatologists chose to de-escalate treatment in either patient they were offered a second question to select the strategy they most preferred. If de-escalation was deemed appropriate, a second choice was given on how they would de-escalate DMARDs. To make respondents familiar with the concept of DCE, two introductory questions were included. To avoid bias by presentation, the order of choice sets and order of attributes were randomized for each participant [13]. An English translation of a complete questionnaire was included (Additional file 4).

### Study sample

All rheumatologists and rheumatology trainees active in the Netherlands were eligible for participation.

### Invitation of subjects

A list of active rheumatologists was kindly provided by the Dutch Society of Rheumatology (NVR). Doctors were recruited during the annual meeting of the NVR on 25 and 26 September 2014. Questionnaires were provided electronically on iPads. Non-attending rheumatologists received an invitation by e-mail to complete the questionnaire at their own computer.

### Statistical analyses

We estimated a conditional logit model to assess overall preference for de-escalation of DMARDs and its determinants (Additional file 5).

To assess whether preferences for de-escalation and relative importance of patient characteristics determining this decision differed among rheumatologists, a subgroup analysis (latent class model) was performed as well (Additional file 3). With this model, subgroups of rheumatologists (clusters or classes) can be identified. To determine the optimal number of classes, we selected the model with the best fit on the consistent Akaike information criterion (cAIC). This measure deals with the trade-off between increase in goodness of fit of the model and the increase in complexity by the addition of clusters. Analyses were performed using the clogit and lclogit function in STATA (version 13.1, StataCorp, College Station, TX, USA).

## Results

In total 156 doctors completed the questionnaire (128 rheumatologists and 28 trainees), 128 of which completed the questionnaire at the annual conference Of 174 rheumatologists that had not participated at the annual conference and received an invitation by e-mail, 28 (16%) completed the questionnaire online. Eleven rheumatologists did not provide demographic data because of technical problems or by their own wishes. Characteristics of the study sample are shown in Table 2.

**Table 2:** Characteristics of study sample

	Rheumatologists (*n* = 117)^a^	Trainee (*n* = 28)
Age (years), median (IQR)	47 (40–57)	34 (31–36)
Female, n (%)	60 (51%)	20 (71%)
Work experience (years), median (IQR)	10 (5–23)	-
Self-reported number of RA patients in practice, median (IQR)	350 (200–1000)	70 (25–100)
Self-reported prevalence of biological treatment among RA patients in practice, median (IQR)	25 (20–30)	30 (20–35)
Working in academic hospital, n (%)	19 (16%)	10 (36%)

^a^Eleven out of 128 rheumatologists did not provide information due to technical problems or by their own wish

De-escalation of therapy was preferred in 74% of the patient choice sets evaluated by the rheumatologists. To quantify how patient characteristics influence doctor’s preferences for de-escalating DMARDs, a conditional logit model was estimated (Table 3). The interpretation of such a model is somewhat different than of a linear or logit regression model. Each of the patient characteristics played a role in the decision to de-escalate DMARDs, as all coefficients of the model showed significance. The opt-out option was chosen if a rheumatologist deemed neither patient of a pair suitable for treatment de-escalation. De-escalation was chosen in 74% of cases, while the opt-out was chosen in 26% of cases. Therefore, in general, the opt-out was less preferred than de-escalation (the reference category), which is reflected by a negative sign for the opt-out (Table 1). Coefficients for the other factors have a similar interpretation, as will be further explained.

**Table 3:** Overall preference of doctors for de-escalation based on a conditional logit model

	Overall *n* = 156	
	β	SE
Opt out^1^ chosen	26%	
Opt out^1^	-2.96^***^	0.11
DAS ≤ 3.2^2^	-0.98^***^	0.07
Swollen joint count		
1^3^	-1.15^***^	0.08
2^3^	-1.68^***^	0.09
Patient history		
Erosive disease^4^	-0.69^***^	0.11
Remission difficult^4^	-0.80^***^	0.09
Erosive + remission difficult^4^	-1.64^***^	0.10
Remission duration 6 months^5^	-0.52^***^	0.06
Patient not willing to de-escalate at start of visit^6^	-1.09^***^	0.07

*β* beta coefficient, *SE* standard error, *DAS* Disease Activity Score

^***^ *p* < 0.001

^1^This option was included in case a rheumatologist did not want to de-escalate DMARDs in either of the patients presented in a pair

^2^Reference DAS < 2.6

^3^Reference no swollen joints

^4^Reference easy remission and no erosions

^5^Reference remission duration 1 year

^6^Reference patient willing to de-escalate DMARDs at start of visit

For each characteristic, we chose the level we expected to be most preferable for de-escalation to be the reference level, e.g., no swollen joints, Disease Activity Score (DAS) < 2.6 (see footnotes Table 3). Therefore, the ideal patient that doctors would like to consider for de-escalation of DMARDs is a patient having all the reference characteristics, i.e., came in remission easily, has no erosions, a remission duration of 1 year, no swollen joints, and has DAS remission rather than DAS LDA. Hence, the coefficients represent the relative decrease in de-escalation preference when a patient presents with that feature (e.g., two swollen joints) relative to the reference of that feature (no swollen joints). Presence of two swollen joints (-1.68, *p* < 0.001) and a patient history of erosive disease in combination with difficulties achieving remission (-1.64, *p* < 0.001) had the strongest influence on the decision not to de-escalate (Table 3). It should be emphasized that above results did not depend on the fashion in which DMARDs were de-escalated, as this was not specified in the questionnaire. Only after rheumatologists chose to de-escalate treatment in either patient they were offered a second question to select the strategy they most preferred (see below).

**Fig. 1:**
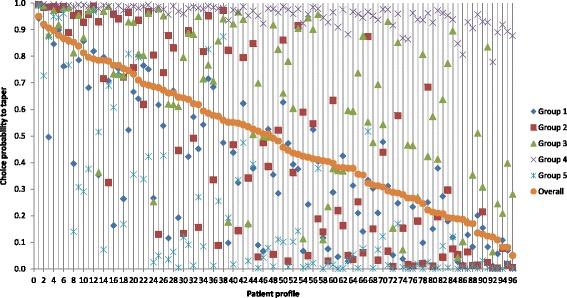
The probability rheumatologists choose to taper specific patients, shown for 96 unique patient profiles. Overall probability (average for all rheumatologists, *orange rounds*) and probabilities by subgroups (*other shapes*) are shown

Although this model describes the preferences of rheumatologists for de-escalation of DMARDs on average, in reality subgroups of doctors may exist that weigh patient characteristics differently. Therefore we looked whether we could distinguish subgroups based on answering patterns. Using a latent class model, we identified five subgroups of rheumatologists as shown in Table 4. Each of the groups made different trade-offs in whom to de-escalate DMARDs given the relative weight in the patient characteristics and opt-out. Opt-out preference ranged from 1% in class 4 to 53% in class 5. For each subgroup the relative weights of the patient characteristics that drove de-escalation were analyzed (absolute weights of coefficients cannot be directly compared between subgroups because coefficients are on different scales). Table 4 shows that presence of swollen joints was the most important characteristic for rheumatologists to consider in subgroups 2 and 5, whereas in subgroups 1 and 3 patient history was most important. Of note, in subgroup 4 none of the patient characteristics dominated the decision to de-escalate, while their preference for de-escalation was strong. To further illustrate differences between the subgroups, 96 unique patient profiles were created, using the characteristics and levels as defined in Table 1. As shown in Fig. 1, overall, patients 1–10 had a high probability to be considered for de-escalation (>80%) and patients 83–96 had a low probability (<20%). For the other patients (11–82), probabilities to be considered for de-escalation were variable among subgroups. Comparing subgroups with respect to their willingness to de-escalation, group 4 had the greatest willingness to de-escalate and group 5 was least willing to de-escalate medication. Given the diagonal in Fig. 1, group 3 had above average willingness to de-escalate, whereas group 1 was somewhat below average. Willingness to de-escalate for group 2 was in general above average for patients at the left side of the figure (patients 1–48) and less than average for patients at the right side (patients 49–96). A simple analysis to see whether differences related to age, sex or practice-related factors (Table 5) did not reveal particular characteristics of doctors. Being a trainee was associated with lower preference to de-escalate.

**Table 4:** Preference of doctors for de-escalation of DMARDs by subgroups based on answering patterns

	Group 1 *n* = 48		Group 2 *n* = 22		Group 3 *n* = 26		Group 4 *n* = 30		Group 5 *n* = 30	
	β	SE	β	SE	β	SE	β	SE	β	SE
Opt-out^1^ chosen	38%		21%		9%		1%		53%	
Opt-out^1^	-2.78^***^	0.23	-5.33^***^	0.97	-4.98^***^	0.83	-5.70^***^	0.64	-4.72^***^	0.41
DAS ≤ 3.2^2^	-0.59^***^	0.15	-1.60^**^	0.49	-2.55^***^	0.57	-0.59^***^	0.15	-1.87^***^	0.27
Swollen joint count										
1^3^	-1.01^***^	0.19	-3.24^***^	0.59	-1.02	0.53	-0.79^***^	0.19	-3.53^***^	0.48
2^3^	-1.90^***^	0.22	-6.06^***^	0.99	-0.63^*^	0.28	-1.25^***^	0.21	-3.92^***^	0.51
Patient history										
Erosive disease^4^	-1.27^***^	0.20	-0.73	0.40	-1.23^**^	0.38	-0.58^*^	0.24	-1.29^***^	0.43
Remission difficult^4^	-1.08^***^	0.20	-0.95^*^	0.46	-1.42^**^	0.42	-0.44	0.27	-1.79^***^	0.37
Erosive + remission difficult^4^	-2.80^***^	0.25	-1.14	0.62	-2.99^***^	0.60	-1.29^***^	0.30	-2.79^***^	0.40
Remission duration 6 months^5^	-0.41^*^	0.16	-1.17^***^	0.40	-0.52	0.31	-0.17	0.14	-3.74	-
Patient not willing to de-escalate at start of visit^6^	-1.61^***^	0.20	-1.05^***^	0.30	-0.98^**^	0.30	-1.14^***^	0.13	-1.49^***^	0.27
Class probabilities, median (range)	0.99 (0.55 – 0.99)		0.99 (0.55 – 0.99)		0.94 (0.58 – 0.99)		0.99 (0.77 – 0.99)		0.98 (0.57 – 0.99)	

*β* beta coefficient, *SE* standard error, *DAS* Disease Activity Score

^*^ *p* < 0.05; ^**^ *p* < 0.01; ^***^ *p* < 0.001

^1^This option was included in case a rheumatologist did not want to de-escalate DMARDs in either of the patients presented in a pair

^2^Reference DAS < 2.6

^3^Reference no swollen joints

^4^Reference easy remission and no erosions

^5^Reference remission duration 1 year

^6^Reference patient willing to de-escalate DMARDs at start of visit

**Table 5:** Characteristics of subgroups

	Group 1 *n* = 48	Group 2 *n* = 22	Group 3 *n* = 26	Group 4 *n* = 30	Group 5 *n* = 30	*p* ^*^
Age (years), median (IQR)	43 (35–51)	49 (40–56)	39 (35–45)	50 (40–60)	41 (37–52)	0.038
Female, n (%)	22 (51%)	12 (55%)	15 (60%)	15 (50%)	20 (67%)	0.663
Trainee, n (%)	11 (26%)	2 (9%)	7 (29%)	1 (3%)	7 (26%)	0.051
Work experience (years), median (IQR)	7 (0–15)	13 (5–25)	5 (0–8)	12 (5–25)	4 (0–18)	0.027
Self-reported number of RA patients in practice, median (IQR)	375 (100–800)	425 (200–1100)	450 (100–2000)	300 (200–1000)	300 (100–500)	0.492
Self-reported prevalence of biological treatment among RA patients in practice, median (IQR)	23 (20–30)	20 (20–33)	30 (20–30)	29 (20–33)	20 (15–30)	0.641
Working in academic hospital, n (%)	9 (21%)	4 (18%)	6 (24%)	5 (17%)	7 (23%)	0.955
No data, n (%)	5 (10%)	0 (0%)	2 (8%)	1 (3%)	3 (10%)	<0.001

*RA* rheumatoid arthritis

^*^Kruskall-Wallis test

After the decision whether to de-escalate or not, the rheumatologists were presented with the choice which DMARDs to de-escalate. Three options were presented: de-escalating the TNF blocker, de-escalating methotrexate (MTX) or de-escalating MTX to half the dose followed by de-escalating the TNF blocker. Of de-escalation strategies, rheumatologists chose de-escalating the TNF blocker in the majority of cases (61%), followed by de-escalating the TNF blocker after de-escalating MTX to half dosage (33%).

## Discussion

By semi-structured interviews, we identified five patient characteristics rheumatologists take into account in their decision to de-escalate DMARDs: number of swollen joints, presence of DAS remission/LDA, patient history, duration of remission/LDA and patient willingness to de-escalate. Using a discrete choice experiment among Dutch rheumatologists, number of swollen joints and patient history were identified as factors of greatest importance. However, rheumatologists were not uniform in their decision to de-escalate DMARDs. Based on a further (latent class) analysis of the answering patterns, five subgroups of rheumatologists were identified that traded off patient characteristics differently in their decision to de-escalate: (1) rheumatologists that always tapered, (2) rheumatologists tapering in the absence of swollen joints, (3) rheumatologists tapering in the absence of swollen joints and in the presence of a favorable patient history, (4) rheumatologists tapering in case of DAS remission and favorable patient history, and (5) rheumatologists taking into account all factors. That heterogeneity among rheumatologists exists with respect to decision-making was further demonstrated by calculating the probability rheumatologists would decide to de-escalate medication for 96 unique patient profiles (Fig. 1). This showed that subgroup probabilities were highly variable for most of the profiles, especially those for which one or more characteristics were less favorable (e.g., presence of one rather than no swollen joints or DAS LDA rather than DAS remission). This means that no general consensus exists on which patients are suitable for de-escalation. Eliciting details on rheumatologist subgroups resulted in mixed demographic characteristics, so other person-related factors are likely to play a role. We only observed that trainees were less willing to taper, possibly due to lack of experience and confidence. Of de-escalation strategies rheumatologists could choose from, de-escalating the TNF blocker first and de-escalating the TNF blocker after reducing MTX to half dosage were chosen in 94% of cases.

To date there is no standardized way to determine the patient for whom de-escalation of DMARD therapy is appropriate [8]. Of clinical factors, conflicting results have been reported for deeper remission [14, 15] and shorter disease duration [14–16] to be associated with successful tapering, while observations from the CORRONA registry suggested that a rapid response to DMARDs is associated with better maintenance of remission when the agents are tapered later on [8, 17]. In this DCE, rheumatologists regarded presence of swollen joints, a patient history of erosive disease and difficulties achieving remission and patient fulfilling DAS LDA rather than remission as most important factors to not de-escalate. Although swollen joints may be regarded as a direct indication of inflammation and contraindication for de-escalation, this is not necessarily true for the DAS28 score itself, which could be high because of psychosocial distress or comorbidities. Rheumatologists in clinical practice may therefore sometimes decide to de-escalate in case of DAS LDA in the absence of other signs of inflammation. Future research may aid to clarify which clinical factors are really important for predicting successful de-escalation. Ultrasound [18–20] or biomarkers [14, 21] may have an additional role in detecting in which patients subclinical synovitis is still present increasing the risk of flare after treatment withdrawal. It would also be of interest to study whether (a combination of) clinical, ultrasonographic and/or serum factors can adequately predict which patients can successfully de-escalate treatment.

This study was conducted using a DCE, which in comparison to other quantified preference techniques, bears most resemblance to real-world decision-making [22]. A strength of this design is that several patient characteristics can be evaluated at once in which the weight of each characteristic contributes to the decision to de-escalate medication. Another strength of this study is that we identified relevant characteristics using semi-structured interviews with a random sample of rheumatologists to identify relevant factors (characteristics and levels) for the DCE questionnaire. As no new factors were mentioned during the final interviews, we assumed that saturation was reached and hence no important factors had been missed. One inherent limitation of a DCE is that rheumatologists were asked to evaluate virtual RA patients on screen. Consequently, as the decision to de-escalate does not have real clinical implications, rheumatologists may have been more risk-taking than they would be when dealing with real patients. This could then have resulted in an overestimation of rheumatologists’ willingness to de-escalate. Another limitation of this study is that, although the majority of rheumatologists attending the annual conference participated in the study, response rates were low for rheumatologists not attending the conference that were invited to participate from home. Although this could for a large part be explained by the method of recruitment, we could not fully exclude the possibility of a relationship between non-responders and tendency to de-escalate DMARDs.

In designing the questions for the DCE we made several choices that need clarification. We refrained from including side effects in the decision to de-escalate. Several rheumatologists remarked that presence of side effects is relevant in the decision of de-escalation therapy. Although we agree, this is likely to influence the decision to de-escalate a particular medicine first due to side effects, it not necessarily relates to the decision of de-escalation in patients achieving LDA or remission. The presence of severe side effects would likely result in de-escalation or switching medication before sustained remission or LDA is achieved.

Second, a simple choice was given on what to de-escalate. Rheumatologists could choose between de-escalating the TNF inhibitor or MTX completely, or to de-escalate the TNF inhibitor completely after reducing MTX to half dosage. As more strategies are imaginable, different strategies can and will be adopted in reality. Therefore, more work on preference of what to de-escalate first given both the medication characteristics and patient characteristics would help to further understand de-escalation of therapy decisions.

Third, due to the definition of levels assigned to the disease characteristics, the relative importance of characteristics could change if levels had been defined differently (e.g., remission duration of 2 years rather than 1 year). Therefore, the relative importance of characteristics can only be interpreted taking the definition of the levels into consideration.

## Conclusions

Swollen joint count (SJC) and patient history were the most important characteristics rheumatologists take into consideration in the decision to de-escalate. However rheumatologists are not uniform in their decision on whom to de-escalate. Five subgroups of rheumatologists could be identified: those that taper (1) always, (2) in absence of swollen joints, (3) in absence of swollen joints and favorable patient history, (4) if DAS remission and favorable patient history, and (5) taking into account all factors.

To improve uniform decision-making in the future, more research is needed assessing the predictive value of patient characteristics for successful de-escalation of DMARDs.

## Additional files

Additional file 1: Topics for semi-structured interview. List of topics that were discussed in the semi-structured interviews. (DOCX 18 kb)

Additional file 2: Priors for the levels of patient characteristics. Priors used in the design of the questionnaire. (DOCX 24 kb)

Additional file 3: Choice set example. Example of a choice set as presented in the questionnaire. (DOCX 23 kb)

Additional file 4: English translation of DCE questionnaire (questionnaire was taken in Dutch). English translation of a complete DCE questionnaire. The original questionnaire was taken in Dutch. (DOCX 89 kb)

Additional file 5: Utility functions for the conditional logit and latent class models. (DOCX 23 kb)

## Abbreviations

ACR: American College of Rheumatology
cAIC: Consistent Akaike Information Criterion
DAS: Disease Activity Score
DCE: Discrete Choice Experiment
DMARDs: Disease-modifying anti-rheumatic drugs
EULAR: European League Against Rheumatism
LDA: Low disease activity
MTX: Methotrexate
NVR: Nederlandse Vereniging voor Reumatologie (Dutch Society of Rheumatology)
RA: Rheumatoid arthritis
SJC: Swollen joint count
TNF: Tumor necrosis factor
