# Supplementary material for: Doctors’ preferences in de-escalating DMARDs in rheumatoid arthritis: a discrete choice experiment
Source: Arthritis Res Ther. 2017 Apr 26;19:78. doi: 10.1186/s13075-017-1287-z (PMC5405491; doi:10.1186/s13075-017-1287-z)
Supplement: Supplementary file 1 — Topics for semi-structured interview. List of topics that were discussed in the semi-structured interviews. (DOCX 18 kb) [file 13075_2017_1287_MOESM1_ESM.docx]

**Additional file 1: Topics for semi-structured interview**

**Introduction:**

Thank you for cooperating with this interview.

We would like to get ourselves an idea about how rheumatologists de-escalate DMARDs in clinical practice in patients with RA.

The following questions will focus on the de-escalation of conventional and biological DMARDs in patients with RA in remission.

Topics to discuss:

- Age/gender/years of clinical practice of rheumatologist
- Have you ever de-escalated biological or conventional DMARDs?
- What are, in your opinion, advantages of de-escalation?
- And disadvantages?
- Which factors make that you consider treatment de-escalation for a certain patient?

(first have rheumatologist come with an answer by himself, thereafter check whether any of below topics are relevant as well)

- - - **Disese duration of RA**
    - **Severity and course of disease**
    - **DMARD history**
    - **“Difficulties” to achieve remission in a patient**
    - **Remission duration**
    - **Doctor-patient relationship (mutual trust, extent to which shared decision making is possible**
    - **Other**
  - Do you sometimes de-escalate medication for a patient for whom it was difficult to achieve a state of remission.
  - In your opinion, how long should a patient be in remission before one should consider tapering or stopping medication?
  - How do you evaluate whether a patient is in remission and can commence de-escalation (DAS, other factors?)
  - If you were to assess remission exclusively by using the DAS28, then which value of the DAS28 would, in your opinion, reflect remission? (Officially 2.6, but especially the feeling of the doctor is relevant here)
  - In clinical practice, the DAS is not always a perfect measure to assess remission. Are there perhaps components of the DAS you feel are a decisive factor to indicate remission.
  - In case you de-escalate etanercept (Enbrel) in patients that use the regular dose (50 mg/ week), then in what fashion do you generally do this? (extending the dose interval, half the dose, stop immediately).
  - Are you aware that etanercept (Enbrel) is also available as a 25 mg injection? Do you sometimes prescribe it?
  - Which would have your preference? De-escalating etanercept (Enbrel) by doubling the interval or by halving the dose by prescribing the 25 mg / week injection?
  - Whenever you de-escalate methotrexate in patients using the full dose (20-25 mg/week), how do you usually do this? (half the dosage at once, taper by 1-2 tablets each time? Stop at once?)
  - In case a patient uses the combination of etanercept (Enbrel) and methotrexate, do you have a preference which agent to de-escalate (first)? How would you de-escalate?
  - What makes that you have a preference?
    - **Adverse effects**
    - **Co-morbidity**
    - **Preference of the patient**
    - **Other**
  - In case a patient uses the combination of adalimumab (Humira) and methotrexate, do you have a preference which agent to de-escalate first? Why? (does the formation of anti-drug antibodies play a role?) How would you de-escalate?
  - In case a patient uses another TNF blocker (certolizumab (Cimzia) 200mg/2 weeks or golimumab (Simponi) 50mg/month), how would you commence de-escalation? (would you then use another de-escalation strategy?)
- Can you imagine that you would decide not to de-escalate medication in a patient that has reached a state of low disease activity or remission for a long period of time? What are the reasons?
- Are there any non-medical reasons that play a role in your decision to de-escalate, such as biological costs or branch recommendations?
- Are there patients whom you never de-escalate after negotiation?
- How many patients do you estimate are in your practice and how many use a biological?
