# Supplementary material for: Doctors’ preferences in de-escalating DMARDs in rheumatoid arthritis: a discrete choice experiment
Source: Arthritis Res Ther. 2017 Apr 26;19:78. doi: 10.1186/s13075-017-1287-z (PMC5405491; doi:10.1186/s13075-017-1287-z)
Supplement: Supplementary file 2 — Priors for the levels of patient characteristics. Priors used in the design of the questionnaire. (DOCX 24 kb) [file 13075_2017_1287_MOESM2_ESM.docx]

Additional file 2: Priors for the levels of patients characteristics

| **Characteristic** | **Levels** | **Priors** |
| --- | --- | --- |
| Duration of remission | - 1 year - 6 months | 0 (ref) -0.1 |
| Patient preference for tapering at the start of the consult | - Patient is not willing to taper - Patient is willing to taper | 0.15 0 (ref) |
| Number of swollen joints | - 0 - 1 - 2 | 0.2 0.1 0 (ref) |
| DAS28 | - < 2.6 - ≤ 3.2 | 0.1 0 (ref) |
| Medical history | - Difficult to accomplish remission - Easy to accomplish remission | -0.1  0 (ref) |
|  | - Non-erosive - Erosive | 0 (ref) -0.15 |
