# Supplementary material for: Doctors’ preferences in de-escalating DMARDs in rheumatoid arthritis: a discrete choice experiment
Source: Arthritis Res Ther. 2017 Apr 26;19:78. doi: 10.1186/s13075-017-1287-z (PMC5405491; doi:10.1186/s13075-017-1287-z)
Supplement: Supplementary file 3 — Choice set example. Example of a choice set as presented in the questionnaire. (DOCX 23 kb) [file 13075_2017_1287_MOESM3_ESM.docx]

**Additional file 3: Choice set example**

|  | **Patient A** | **Patient B** | **Opt-out** |
| --- | --- | --- | --- |
| Duration of remission | 1 year | 6 months | - |
| Patient preference for tapering at start of visit | Patient is not willing to taper | Patient is willing to taper | - |
| Number of swollen joints | 1 | 0 | - |
| DAS28 | < 2.6 | ≤ 3.2 | - |
| Medical history | Difficult to accomplish remission  Non-erosive | Easy to accomplish remission | - |
|  |  |  |  |
| **In case A or B was chosen:** | **Strategy A** | **Strategy B** | **Strategy C** |
| Preferred tapering strategy | Tapering MTX to 0 mg | Tapering biological to 0 mg | Decrease MTX (50% of initial dosage), then taper biological to 0 mg |
