# Supplementary material for: Doctors’ preferences in de-escalating DMARDs in rheumatoid arthritis: a discrete choice experiment
Source: Arthritis Res Ther. 2017 Apr 26;19:78. doi: 10.1186/s13075-017-1287-z (PMC5405491; doi:10.1186/s13075-017-1287-z)
Supplement: Supplementary file 4 — English translation of DCE questionnaire (questionnaire was taken in Dutch). English translation of a complete DCE questionnaire. The original questionnaire was taken in Dutch. (DOCX 89 kb) [file 13075_2017_1287_MOESM4_ESM.docx]

**Additional file 4: English translation of DCE questionnaire (questionnaire was taken in Dutch)**

Dear rheumatologist,

Thank you for participating in this study! Below you can read a short explanation on what a DCE is.

A DCE is a questionnaire in which various scenarios are presented. Every time you will need to choose from two scenarios the one you deem most suited for the decision you need to make. By analyzing the choices rheumatologists make with respect to the scenarios we can measure and evaluate the rheumatologists’ preferences with respect to de-escalation in clinical practice.

First you will get 2 example questions.

This questionnaire contains 41 questions.

**Example question 1**

**[]**

**Which destination for vacation would you choose?**

|  | **A** | **B** |
| --- | --- | --- |
| Destination | Tasting wine in France | Tour around the USA |
| Accomodation | Chalet | Hotels |
| Probability for sunny weather | 60% | 90% |
| Days | 14 | 21 |
| Costs per person | € 1500,- | €3000,- |

*****

|  | Destination A | Destination B |
| --- | --- | --- |
| Which one would you prefer? | 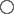 | 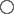 |

**Example question 2**

**[]**

**Which mode of transportation would you prefer?**

|  | **Car** | **Bus** |
| --- | --- | --- |
| **Duration of travelling time** | **20 min** | **25 min** |
| **Time to reach transport** | **0 min** | **3 min** |
| **Costs** | **€ 10** | **€ 4** |

*****

|  | Car | Bus |
| --- | --- | --- |
| Which one would you prefer? | 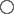 | 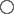 |

**All patients mentioned below use the combination of methotrexate (20-25 mg) with a TNF-blocker.**

**Choice task 1**

**[]**

**In which patient would you de-escalate treatment?**

|  | **Patient A** | **Patient B** |
| --- | --- | --- |
| **Duration of remission** | 6 months | 1 year |
| **Patient preference for de-escalation at the start of the consult** | Patient is not willing to de-escalate | Patient is willing to de-escalate |
| **Number of swollen joints** | 1 | 2 |
| **DAS28** | ≤ 3.2 | < 2.6 |
| **Medical history** | Difficult to accomplish remission | Easy to accomplish remission |
|  | Non-erosive | Erosive |

*****

|  | Scenario A | Scenario B | Neither |
| --- | --- | --- | --- |
| Choice | 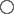 | 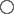 | 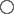 |

**[]**

**Which agent would you like to de-escalate if the selected patient were using methotrexate and a TNF-blocker?**

| **Option A** | **Option B** | **Option C** |
| --- | --- | --- |
| De-escalate methotrexate to 0mg | De-escalate the TNF-blocker to 0mg | First half methotrexate, then de-escalate the TNF-blocker to 0mg |

*****

|  | Option A | Option B | Option C |
| --- | --- | --- | --- |
| Choice | 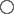 | 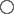 | 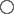 |

**All patients mentioned below use the combination of methotrexate (20-25 mg) with a TNF-blocker.**

**Choice task 2**

**[]**

**In which patient would you de-escalate treatment?**

|  | **Patient A** | **Patient B** |
| --- | --- | --- |
| **Duration of remission** | 1 year | 6 months |
| **Patient preference for de-escalation at the start of the consult** | Patient is willing to de-escalate | Patient is not willing to de-escalate |
| **Number of swollen joints** | 1 | 2 |
| **DAS28** | ≤ 3.2 | < 2.6 |
| **Medical history** | Easy to accomplish remission | Difficult to accomplish remission |
|  | Non erosive | Erosive |

*****

|  | Scenario A | Scenario B | Neither |
| --- | --- | --- | --- |
| Choice | 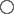 | 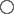 | 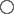 |

**[]**

**Which agent would you like to de-escalate if the selected patient were using methotrexate and a TNF-blocker?**

| **Option A** | **Option B** | **Option C** |
| --- | --- | --- |
| De-escalate methotrexate to 0mg | De-escalate the TNF-blocker to 0mg | First half methotrexate, then de-escalate the TNF-blocker to 0mg |

*****

|  | Option A | Option B | Option C |
| --- | --- | --- | --- |
| Choice | 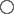 | 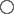 | 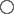 |

**All patients mentioned below use the combination of methotrexate (20-25 mg) with a TNF-blocker.**

**Choice task 3**

**[]**

**In which patient would you de-escalate treatment?**

|  | **Patient A** | **Patient B** |
| --- | --- | --- |
| **Duration of remission** | 6 months | 1 year |
| **Patient preference for de-escalation at the start of the consult** | Patient is not willing to de-escalate | Patient is willing to de-escalate |
| **Number of swollen joints** | 2 | 0 |
| **DAS28** | < 2.6 | ≤ 3.2 |
| **Medical history** | Easy to accomplish remission | Difficult to accomplish remission |
|  | Erosive | Erosive |

*****

|  | Scenario A | Scenario B | Neither |
| --- | --- | --- | --- |
| Choice | 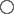 | 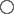 | 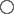 |

**[]**

**Which agent would you like to de-escalate if the selected patient were using methotrexate and a TNF-blocker?**

| **Option A** | **Option B** | **Option C** |
| --- | --- | --- |
| De-escalate methotrexate to 0mg | De-escalate the TNF-blocker to 0mg | First half methotrexate, then de-escalate the TNF-blocker to 0mg |

*****

|  | Option A | Option B | Option C |
| --- | --- | --- | --- |
| Choice | 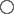 | 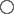 | 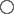 |

**All patients mentioned below use the combination of methotrexate (20-25 mg) with a TNF-blocker.**

**Choice task 4**

**[]**

**In which patient would you de-escalate treatment?**

|  | **Patient A** | **Patient B** |
| --- | --- | --- |
| **Duration of remission** | 6 months | 1 year |
| **Patient preference for de-escalation at the start of the consult** | Patient is willing to de-escalate | Patient is not willing to de-escalate |
| **Number of swollen joints** | 2 | 0 |
| **DAS28** | < 2.6 | ≤ 3.2 |
| **Medical history** | Difficult to accomplish remission | Easy to accomplish remission |
|  | Erosive | Erosive |

*****

|  | Scenario A | Scenario B | Neither |
| --- | --- | --- | --- |
| Choice | 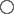 | 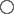 | 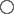 |

**[]**

**Which agent would you like to de-escalate if the selected patient were using methotrexate and a TNF-blocker?**

| **Option A** | **Option B** | **Option C** |
| --- | --- | --- |
| De-escalate methotrexate to 0mg | De-escalate the TNF-blocker to 0mg | First half methotrexate, then de-escalate the TNF-blocker to 0mg |

*****

|  | Option A | Option B | Option C |
| --- | --- | --- | --- |
| Choice | 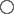 | 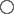 | 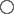 |

**All patients mentioned below use the combination of methotrexate (20-25 mg) with a TNF-blocker.**

**Choice task 5**

**[]**

**In which patient would you de-escalate treatment?**

|  | **Patient A** | **Patient B** |
| --- | --- | --- |
| **Duration of remission** | 6 months | 1 year |
| **Patient preference for de-escalation at the start of the consult** | Patient is willing to de-escalate | Patient is not willing to de-escalate |
| **Number of swollen joints** | 2 | 0 |
| **DAS28** | ≤ 3.2 | < 2.6 |
| **Medical history** | Easy to accomplish remission | Difficult to accomplish remission |
|  | Erosive | Erosive |

*****

|  | Scenario A | Scenario B | Neither |
| --- | --- | --- | --- |
| Choice | 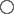 | 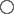 | 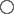 |

**[]**

**Which agent would you like to de-escalate if the selected patient were using methotrexate and a TNF-blocker?**

| **Option A** | **Option B** | **Option C** |
| --- | --- | --- |
| De-escalate methotrexate to 0mg | De-escalate the TNF-blocker to 0mg | First half methotrexate, then de-escalate the TNF-blocker to 0mg |

*****

|  | Option A | Option B | Option C |
| --- | --- | --- | --- |
| Choice | 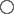 | 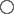 | 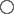 |

**All patients mentioned below use the combination of methotrexate (20-25 mg) with a TNF-blocker.**

**Choice task 6**

**[]**

**In which patient would you de-escalate treatment?**

|  | **Patient A** | **Patient B** |
| --- | --- | --- |
| **Duration of remission** | 6 months | 1 year |
| **Patient preference for de-escalation at the start of the consult** | Patient is willing to de-escalate | Patient is not willing to de-escalate |
| **Number of swollen joints** | 1 | 2 |
| **DAS28** | ≤ 3.2 | < 2.6 |
| **Medical history** | Difficult to accomplish remission | Difficult to accomplish remission |
|  | Erosive | Non erosive |

*****

|  | Scenario A | Scenario B | Neither |
| --- | --- | --- | --- |
| Choice | 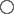 | 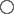 | 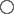 |

**[]**

**Which agent would you like to de-escalate if the selected patient were using methotrexate and a TNF-blocker?**

| **Option A** | **Option B** | **Option C** |
| --- | --- | --- |
| De-escalate methotrexate to 0mg | De-escalate the TNF-blocker to 0mg | First half methotrexate, then de-escalate the TNF-blocker to 0mg |

*****

|  | Option A | Option B | Option C |
| --- | --- | --- | --- |
| Choice | 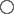 | 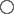 | 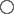 |

**All patients mentioned below use the combination of methotrexate (20-25 mg) with a TNF-blocker.**

**Choice task 7**

**[]**

**In which patient would you de-escalate treatment?**

|  | **Patient A** | **Patient B** |
| --- | --- | --- |
| **Duration of remission** | 1 year | 6 months |
| **Patient preference for de-escalation at the start of the consult** | Patient is willing to de-escalate | Patient is not willing to de-escalate |
| **Number of swollen joints** | 0 | 2 |
| **DAS28** | < 2.6 | ≤ 3.2 |
| **Medical history** | Easy to accomplish remission | Easy to accomplish remission |
|  | Erosive | Non erosive |

*****

|  | Scenario A | Scenario B | Neither |
| --- | --- | --- | --- |
| Choice | 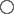 | 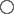 | 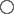 |

**[]**

**Which agent would you like to de-escalate if the selected patient were using methotrexate and a TNF-blocker?**

| **Option A** | **Option B** | **Option C** |
| --- | --- | --- |
| De-escalate methotrexate to 0mg | De-escalate the TNF-blocker to 0mg | First half methotrexate, then de-escalate the TNF-blocker to 0mg |

*****

|  | Option A | Option B | Option C |
| --- | --- | --- | --- |
| Choice | 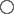 | 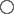 | 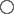 |

**All patients mentioned below use the combination of methotrexate (20-25 mg) with a TNF-blocker.**

**Choice task 8**

**[]**

**In which patient would you de-escalate treatment?**

|  | **Patient A** | **Patient B** |
| --- | --- | --- |
| **Duration of remission** | 1 year | 6 months |
| **Patient preference for de-escalation at the start of the consult** | Patient is willing to de-escalate | Patient is not willing to de-escalate |
| **Number of swollen joints** | 2 | 0 |
| **DAS28** | ≤ 3.2 | < 2.6 |
| **Medical history** | Difficult to accomplish remission | Easy to accomplish remission |
|  | Erosive | Non erosive |

*****

|  | Scenario A | Scenario B | Neither |
| --- | --- | --- | --- |
| Choice | 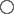 | 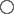 | 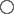 |

**[]**

**Which agent would you like to de-escalate if the selected patient were using methotrexate and a TNF-blocker?**

| **Option A** | **Option B** | **Option C** |
| --- | --- | --- |
| De-escalate methotrexate to 0mg | De-escalate the TNF-blocker to 0mg | First half methotrexate, then de-escalate the TNF-blocker to 0mg |

*****

|  | Option A | Option B | Option C |
| --- | --- | --- | --- |
| Choice | 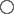 | 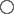 | 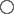 |

**All patients mentioned below use the combination of methotrexate (20-25 mg) with a TNF-blocker.**

**Choice task 9**

**[]**

**In which patient would you de-escalate treatment?**

|  | **Patient A** | **Patient B** |
| --- | --- | --- |
| **Duration of remission** | 1 year | 6 months |
| **Patient preference for de-escalation at the start of the consult** | Patient is not willing to de-escalate | Patient is willing to de-escalate |
| **Number of swollen joints** | 2 | 1 |
| **DAS28** | ≤ 3.2 | < 2.6 |
| **Medical history** | Easy to accomplish remission | Easy to accomplish remission |
|  | Non erosive | Erosive |

*****

|  | Scenario A | Scenario B | Neither |
| --- | --- | --- | --- |
| Choice | 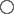 | 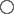 | 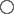 |

**[]**

**Which agent would you like to de-escalate if the selected patient were using methotrexate and a TNF-blocker?**

| **Option A** | **Option B** | **Option C** |
| --- | --- | --- |
| De-escalate methotrexate to 0mg | De-escalate the TNF-blocker to 0mg | First half methotrexate, then de-escalate the TNF-blocker to 0mg |

*****

|  | Option A | Option B | Option C |
| --- | --- | --- | --- |
| Choice | 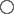 | 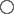 | 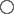 |

**All patients mentioned below use the combination of methotrexate (20-25 mg) with a TNF-blocker.**

**Choice task 10**

**[]**

**In which patient would you de-escalate treatment?**

|  | **Patient A** | **Patient B** |
| --- | --- | --- |
| **Duration of remission** | 6 months | 1 year |
| **Patient preference for de-escalation at the start of the consult** | Patient is willing to de-escalate | Patient is not willing to de-escalate |
| **Number of swollen joints** | 0 | 1 |
| **DAS28** | < 2.6 | ≤ 3.2 |
| **Medical history** | Easy to accomplish remission | Difficult to accomplish remission |
|  | Non erosive | Erosive |

*****

|  | Scenario A | Scenario B | Neither |
| --- | --- | --- | --- |
| Choice | 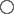 | 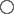 | 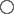 |

**[]**

**Which agent would you like to de-escalate if the selected patient were using methotrexate and a TNF-blocker?**

| **Option A** | **Option B** | **Option C** |
| --- | --- | --- |
| De-escalate methotrexate to 0mg | De-escalate the TNF-blocker to 0mg | First half methotrexate, then de-escalate the TNF-blocker to 0mg |

*****

|  | Option A | Option B | Option C |
| --- | --- | --- | --- |
| Choice | 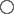 | 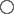 | 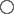 |

**All patients mentioned below use the combination of methotrexate (20-25 mg) with a TNF-blocker.**

**Choice task 11**

**[]**

**In which patient would you de-escalate treatment?**

|  | **Patient A** | **Patient B** |
| --- | --- | --- |
| **Duration of remission** | 6 months | 1 year |
| **Patient preference for de-escalation at the start of the consult** | Patient is not willing to de-escalate | Patient is willing to de-escalate |
| **Number of swollen joints** | 0 | 1 |
| **DAS28** | ≤ 3.2 | < 2.6 |
| **Medical history** | Difficult to accomplish remission | Easy to accomplish remission |
|  | Non erosive | Non erosive |

*****

|  | Scenario A | Scenario B | Neither |
| --- | --- | --- | --- |
| Choice | 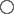 | 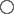 | 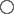 |

**[]**

**Which agent would you like to de-escalate if the selected patient were using methotrexate and a TNF-blocker?**

| **Option A** | **Option B** | **Option C** |
| --- | --- | --- |
| De-escalate methotrexate to 0mg | De-escalate the TNF-blocker to 0mg | First half methotrexate, then de-escalate the TNF-blocker to 0mg |

*****

|  | Option A | Option B | Option C |
| --- | --- | --- | --- |
| Choice | 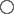 | 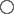 | 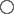 |

**All patients mentioned below use the combination of methotrexate (20-25 mg) with a TNF-blocker.**

**Choice task 12**

**[]**

**In which patient would you de-escalate treatment?**

|  | **Patient A** | **Patient B** |
| --- | --- | --- |
| **Duration of remission** | 1 year | 6 months |
| **Patient preference for de-escalation at the start of the consult** | Patient is willing to de-escalate | Patient is not willing to de-escalate |
| **Number of swollen joints** | 0 | 1 |
| **DAS28** | < 2.6 | ≤ 3.2 |
| **Medical history** | Difficult to accomplish remission | Easy to accomplish remission |
|  | Non erosive | Erosive |

*****

|  | Scenario A | Scenario B | Neither |
| --- | --- | --- | --- |
| Choice | 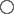 | 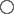 | 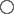 |

**[]**

**Which agent would you like to de-escalate if the selected patient were using methotrexate and a TNF-blocker?**

| **Option A** | **Option B** | **Option C** |
| --- | --- | --- |
| De-escalate methotrexate to 0mg | De-escalate the TNF-blocker to 0mg | First half methotrexate, then de-escalate the TNF-blocker to 0mg |

*****

|  | Option A | Option B | Option C |
| --- | --- | --- | --- |
| Choice | 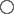 | 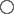 | 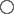 |

**All patients mentioned below use the combination of methotrexate (20-25 mg) with a TNF-blocker.**

**Choice task 13**

**[]**

**In which patient would you de-escalate treatment?**

|  | **Patient A** | **Patient B** |
| --- | --- | --- |
| **Duration of remission** | 6 months | 1 year |
| **Patient preference for de-escalation at the start of the consult** | Patient is not willing to de-escalate | Patient is willing to de-escalate |
| **Number of swollen joints** | 0 | 2 |
| **DAS28** | < 2.6 | ≤ 3.2 |
| **Medical history** | Difficult to accomplish remission | Difficult to accomplish remission |
|  | Erosive | Non erosive |

*****

|  | Scenario A | Scenario B | Neither |
| --- | --- | --- | --- |
| Choice | 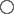 | 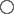 | 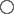 |

**[]**

**Which agent would you like to de-escalate if the selected patient were using methotrexate and a TNF-blocker**

| **Option A** | **Option B** | **Option C** |
| --- | --- | --- |
| De-escalate methotrexate to 0mg | De-escalate the TNF-blocker to 0mg | First half methotrexate, then de-escalate the TNF-blocker to 0mg |

*****

|  | Option A | Option B | Option C |
| --- | --- | --- | --- |
| Choice | 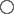 | 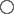 | 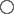 |

**All patients mentioned below use the combination of methotrexate (20-25 mg) with a TNF-blocker.**

**Choice task 14**

**[]**

**In which patient would you de-escalate treatment?**

|  | **Patient A** | **Patient B** |
| --- | --- | --- |
| **Duration of remission** | 1 year | 6 months |
| **Patient preference for de-escalation at the start of the consult** | Patient is not willing to de-escalate | Patient is willing to de-escalate |
| **Number of swollen joints** | 1 | 0 |
| **DAS28** | < 2.6 | ≤ 3.2 |
| **Medical history** | Easy to accomplish remission | Difficult to accomplish remission |
|  | Non erosive | Non erosive |

*****

|  | Scenario A | Scenario B | Neither |
| --- | --- | --- | --- |
| Choice | 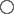 | 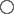 | 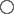 |

**[]**

**Which agent would you like to de-escalate if the selected patient were using methotrexate and a TNF-blocker**

| **Option A** | **Option B** | **Option C** |
| --- | --- | --- |
| De-escalate methotrexate to 0mg | De-escalate the TNF-blocker to 0mg | First half methotrexate, then de-escalate the TNF-blocker to 0mg |

*****

|  | Option A | Option B | Option C |
| --- | --- | --- | --- |
| Choice | 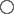 | 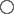 | 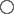 |

**All patients mentioned below use the combination of methotrexate (20-25 mg) with a TNF-blocker.**

**Choice task 15**

**[]**

**In which patient would you de-escalate treatment?**

|  | **Patient A** | **Patient B** |
| --- | --- | --- |
| **Duration of remission** | 1 year | 6 months |
| **Patient preference for de-escalation at the start of the consult** | Patient is not willing to de-escalate | Patient is willing to de-escalate |
| **Number of swollen joints** | 1 | 0 |
| **DAS28** | < 2.6 | ≤ 3.2 |
| **Medical history** | Difficult to accomplish remission | Easy to accomplish remission |
|  | Non erosive | Non erosive |

*****

|  | Scenario A | Scenario B | Neither |
| --- | --- | --- | --- |
| Choice | 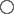 | 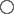 | 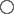 |

**[]**

**Which agent would you like to de-escalate if the selected patient were using methotrexate and a TNF-blocker**

| **Option A** | **Option B** | **Option C** |
| --- | --- | --- |
| De-escalate methotrexate to 0mg | De-escalate the TNF-blocker to 0mg | First half methotrexate, then de-escalate the TNF-blocker to 0mg |

*****

|  | Option A | Option B | Option C |
| --- | --- | --- | --- |
| Choice | 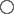 | 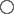 | 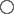 |

**All patients mentioned below use the combination of methotrexate (20-25 mg) with a TNF-blocker.**

**Choice task 16**

**[]**

**In which patient would you de-escalate treatment?**

|  | **Patient A** | **Patient B** |
| --- | --- | --- |
| **Duration of remission** | 1 year | 6 months |
| **Patient preference for de-escalation at the start of the consult** | Patient is not willing to de-escalate | Patient is willing to de-escalate |
| **Number of swollen joints** | 0 | 1 |
| **DAS28** | ≤ 3.2 | < 2.6 |
| **Medical history** | Easy to accomplish remission | Difficult to accomplish remission |
|  | Erosive | Non erosive |

*****

|  | Scenario A | Scenario B | Neither |
| --- | --- | --- | --- |
| Choice | 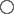 | 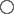 | 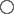 |

**[]**

**Which agent would you like to de-escalate if the selected patient were using methotrexate and a TNF-blocker**

| **Option A** | **Option B** | **Option C** |
| --- | --- | --- |
| De-escalate methotrexate to 0mg | De-escalate the TNF-blocker to 0mg | First half methotrexate, then de-escalate the TNF-blocker to 0mg |

*****

|  | Option A | Option B | Option C |
| --- | --- | --- | --- |
| Choice | 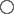 | 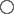 | 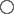 |

**General**

**[]What is your age? ***

**[]Are you a male or a female? ***

|  | Male | Female |
| --- | --- | --- |
| Choice |  |  |

**[]In which type of center do you work at this moment? ***

|  | General hospital | Academic hospital |
| --- | --- | --- |
| Choice |  |  |

**[]**

**How many patients with RA are in your practice? ***

**[]**

**What percentage of RA patients in your practice uses a biological?**

**[]**

**For how many years have you been registered as a rheumatologist?**

**[]**

**In which hospital did you follow your training for rheumatologist?**

*****

Send your questionnaire
Thank you for participating.
