# Supplementary material for: Doctors’ preferences in de-escalating DMARDs in rheumatoid arthritis: a discrete choice experiment
Source: Arthritis Res Ther. 2017 Apr 26;19:78. doi: 10.1186/s13075-017-1287-z (PMC5405491; doi:10.1186/s13075-017-1287-z)
Supplement: Supplementary file 5 — Utility functions for the conditional logit and latent class models. (DOCX 23 kb) [file 13075_2017_1287_MOESM5_ESM.docx]

**Additional file 5**

Utility function for the conditional logit model:

V = β_0_ + β_1_*opt out + β_2_*DAS28≤3.2 + β_3_*SJC=1 + β_4_*SJC=2 + β_5_*history erosive disease + β_6_*history difficult achieving remission + β_7_*history erosive disease and difficult achieving remission + β_8_*remission duration 1 year + β_9_*patient unwilling to taper

V: Observable utility that rheumatologists have for tapering medication in a patient.

β_1_-β_9_: Coefficients for the patient characteristics. These represent the relative weight doctors attach to a certain characteristics when it comes to their decision to taper medication

Utility function for the cluster (latent class) model:

V_|c_ = β_0|c_ + β_1|c_*opt out + β_2|c_*DAS28≤3.2 + β_3|c_*SJC=1 + β_4|c_*SJC=2 + β_5|c_*history erosive disease + β_6|c_*history difficult achieving remission + β_7|c_*history erosive disease and difficult achieving remission + β_8|c_*remission duration 1 year + β_9|c_*patient unwilling to taper

V_|c_: Observable utility for tapering medication for doctors belonging to that class.

β_1_-β_9:_ Represent the coefficients of the attributes indicating the relative weight rheumatologists of a class place on a certain attribute level.
